# The role and potential therapeutic intervention of cellular senescence in intervertebral disc degeneration

**DOI:** 10.1016/j.gendis.2025.101871

**Published:** 2025-09-24

**Authors:** Yunbo Guan, Xuedong Bai, Chao Li, Ziqiang Zhang, Qing He, Lin Chen, Yangli Xie, Zuqiang Wang

**Affiliations:** a Department of Orthopedics, The Sixth Medical Center, General Hospital of Chinese PLA, Beijing 100048, China; b Navy Clinical College, Anhui Medical University, Hefei, Anhui 230000, China; c Department of Wound Repair and Rehabilitation Medicine, State Key Laboratory of Trauma and Chemical Poisoning, Army Medical Center, Daping Hospital, Army Medical University, Chongqing 400038, China

**Keywords:** Autophagy, Cellular senescence, Intervertebraldisc degeneration, Senescence-associated secretory phenotype, Senotherapeutics

## Abstract

Intervertebral disc degeneration (IDD) is a common cause of low back pain that causes significant debilitation. The intricate mechanisms governing intervertebral disc homeostasis are substantially impacted by cellular senescence, which plays crucial roles in both pathological and physiological contexts. In this review, we provide a comprehensive overview of cellular senescence in the pathogenesis of IDD. Specifically, we summarize the merits and limitations of the methodologies utilized in prior investigations to determine cellular senescence and the potential regulatory mechanisms underlying it in the progression of IDD. Furthermore, we describe therapeutic strategies that aim to inhibit cellular senescence, which may alleviate the pathogenesis of IDD. At last, we discuss the challenges and prospects of translational research on targeting cellular senescence in IDD treatment.

## Introduction

**Figure 1:**
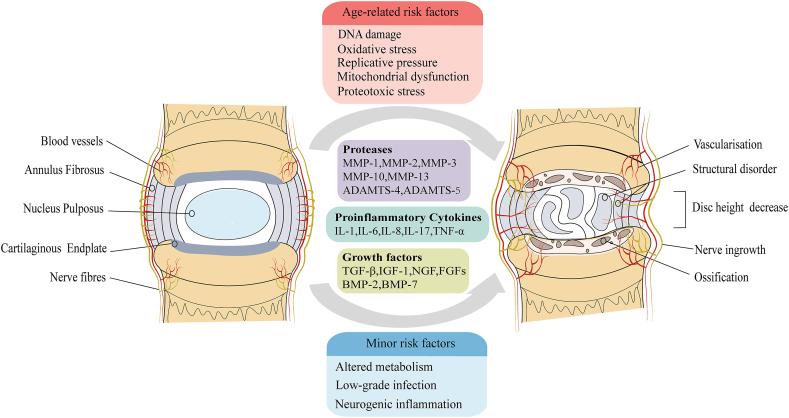
Pathological changes of intervertebral disc degeneration and related risk factors.

The intervertebral disc (IVD) comprises the nucleus pulposus (NP), annulus fibrosus (AF), and cartilaginous endplates (CEPs), which collectively exert a crucial impact on the mobility of the spine.^1^ Among the components of the intervertebral disc, the NP is a highly elastic substance with a jelly-like consistency, marked by a fibrous reticular structure formed by the intersection of chondrocytes and proteoglycan mucoid matrix.^2^ The AF can be categorized into two specific regions, namely the inner and outer AF. Specifically, the inner AF is predominantly composed of type II collagen, derived from rounded fibrocartilage cells, whilst the outer AF primarily consists of slender fibroblasts that are responsible for synthesizing type I collagen.^3^ The CEP predominantly consists of hyaline cartilage cells embedded in extracellular matrix, crucial for maintaining the biomechanical stability of the spine.^4^^,^^5^ Low back pain is a prevalent condition that affects the global population, leading to significant debilitation, with IDD being a main cause.^6^ It can be elicited by various factors and profoundly affects individuals' quality of life, in addition to creating substantial personal and socio-economic challenges.^7^ The occurrence of IDD is the result of complex and multiple risk factors, primarily including genetic predisposition, altered metabolism, low-grade infection, neurogenic inflammation, and aging.^8^, ^9^, ^10^, ^11^, ^12^ Currently, interventions for IDD are limited to early pain relief, rehabilitation treatment, and surgical modalities. The inadequacy of effective treatments for IDD can largely be attributed to a lack of a comprehensive understanding of its underlying mechanisms. Fortunately, the development and research of effective prevention and therapeutic measures for IDD have been given attention by some scholars during their relevant mechanism research. Among these, cellular senescence is widely mentioned. At present, exploring the pathogenesis of cellular senescence in IDD and the causes of cellular damage and death that lead to pathological changes and symptom exacerbation may offer novel insights into IDD management. Furthermore, elucidating the underlying mechanisms that promote cellular senescence could facilitate the development of targeted therapies aimed at rejuvenating senescent cells and restoring their normal functions. Studies have focused on alleviating the clinical symptoms of IDD by targeting the cellular senescence process or by modulating specific signaling pathways, leading to optimized treatment options for patients (Fig. 1).

Cellular senescence refers to a condition in which cells lose their ability to differentiate and function normally. One of its manifestations involves the secretion of various soluble factors in the microenvironment, including proinflammatory cytokines, chemokines, growth factors, and proteases, collectively known as the senescence-associated secretory phenotype (SASP), alongside a permanent halt in cell proliferation.^13^ Throughout human life, senescent cells are consistently produced and play beneficial roles in both physiological and pathological processes, encompassing wound healing and tumor suppression.^14^^,^^15^ Meanwhile, the continuous accumulation of senescent cells over time can also exert detrimental effects. For instance, these senescent cells occupy key cellular ecological niches, contributing to the onset and progression of various skeletal disorders and increasing their morbidity, such as osteoarthritis, osteoporosis, and IDD.^16^, ^17^, ^18^ While aging is related to the occurrence and development of IDD and can lead to intervertebral disc-related cell senescence, its precise mechanism remains elusive.

Anti-senescence therapies present a promising new avenue for the treatment of IDD. Senotherapeutis, a type of anti-senescence drug, selectively targets senescent cells. Indeed, they can regulate senescent cells by inhibiting SASP or eliminating senescent cells via the immune system.^19^ Several studies on targeting aging-related diseases, such as idiopathic pulmonary fibrosis, diabetic macular edema, Alzheimer’s disease, and osteoarthritis, are currently undergoing phase I clinical trials.^20^, ^21^, ^22^ However, the paucity of empirical studies examining the specificity and efficacy of these pharmaceuticals for the management of IDD has resulted in a lack of robust evidence for their application as a clinical intervention strategy.

This review aims to outline the potential mechanisms underlying cellular senescence and its related effects in IDD. Additionally, this review summarizes drug candidates targeting cellular senescence for IDD treatment. These candidates may serve as potential therapeutic approaches to attenuating IDD progression.

### Cellular senescence and SASP

Hayflick and Moorhead’s seminal discovery of cellular senescence in 1961 highlighted it as an irreversible state of cell cycle arrest due to replication stress and aging.^23^ Contemporary research underscores that cellular senescence is not simply a decline in cell division but reflects broader changes in cellular physiology. As cells divide, their chromosomal telomeres become progressively shorter.^24^ When telomeres reach a critical length, cells undergo replicative senescence, entering a viable but permanently arrested state in the cell cycle.^25^ It is well established that senescent cells accumulate as organisms age, particularly in those more susceptible to cellular damage.^26^ In both experimental animals and humans, senescent cell numbers surge in a variety of disease states, including IDD, over time.

A multitude of stressors, such as DNA damage, oncogenic signaling, mitochondrial dysfunction, reactive metabolites, oxidative stress, replication pressure, proteotoxic stress, infection, and harmful factors released by senescent cells into the microenvironment, can induce cellular senescence.^27^, ^28^, ^29^ DNA damage is a primary senescence-inducing stressor, triggering the DNA damage response and activating the well-known p53-p21 and/or p16^INK4a^/retinoblastoma (Rb) pathway.^30^^,^^31^ P21 serves to inhibit the cell cycle by hindering the cyclin-dependent kinase complex, which in turn prevents the formation of the DREAM (dimerization partner, RB-like, E2F, and multi-vulval class B) complex, reducing the expression of cell cycle genes.^32^ In contrast, senescence influenced by epigenetic changes often involves p16, which blocks the formation of the cyclin D–cyclin-dependent kinase 4/6 (D-CDK4/6) complex, leading to the stabilization of the RB-early 2 factor (E2F) complex and subsequent inhibition of cell cycle gene transcription.^33^^,^^34^

Senescent cells within the organism undergo metabolic and physiological transformations through a collection of small-molecule compounds known as SASP. This dynamic process unfolds days after senescence onset. SASP is mechanistically involved in activating nuclear factor kappa B (NF-κB) and associated classical inflammatory regulators. The transcription factor GATA-binding protein 4 (GATA4), which is stabilized in senescent cells, plays a crucial role in SASP regulation. Typically, GATA4 is subject to degradation by P62-mediated selective autophagy. However, studies have shown that post-DNA damage, ataxia-telangiectasia-mutated (ATM) and ataxia telangiectasia and Rad3-related (ATR) activation can disrupt P62-mediated autophagy of GATA4, leading to NF-κB activation through tumor necrosis factor receptor-associated factor 3 (TRAF3)-interacting protein 2 (TRAF3IP2) and interleukin-1α (IL-1α), which promotes SASP and senescence.^35^ Other research suggests that GATA4’s role in DNA damage response signaling becomes prominent only after its accumulation in senescent cells, where it indirectly boosts IL-1α levels, influencing the NF-κB pathway and contributing to SASP production.^36^^,^^37^ Although earlier studies have emphasized different mechanistic pathways, they collectively point to GATA4 as a key molecular switch connecting DNA damage, autophagy, and SASP. Given the discrepancies in GATA’4 involvement in SASP formation and senescence, a comprehensive understanding of its function necessitates an integrated perspective, considering diverse cellular environments and temporal states, so as to further clarify the precise regulatory mechanism of GATA4 under various cell types and pathological conditions in the future. Further research is essential to clarify the mechanisms of cellular senescence and to develop therapeutic strategies for senescence-associated disease.

### Markers of cellular senescence

At present, a challenge facing studies focusing on cellular senescence is the lack of a universal, reliable model-specific marker to accurately identify senescent cells in tissues. Identifying senescent cells in cultured cells or tissue samples generally requires the use of various markers. A positive test for senescence markers reflects that there are senescent cells with permanent growth stagnation in target tissues or cells. Senescent cell exhibits distinct features compared with healthy cells. This section provides a review of commonly used markers and their effectiveness in detecting cellular senescence, including senescence-associated β-galactosidase, P16, P53, and P21 (Table 1).

**Table 1:** Markers of cell senescence.

	Markers of cell senescence	Forms of expression
DNA damage	Telomere	Shortened or attrition
	Cytoplasmic chromatin fragments	Activation of the cGAS-cGAMP-STING pathway-mediated pro-inflammatory response
Protein damage	Reactive oxygen species	Increased production than before
	Phospho-ERK	Higher expression levels than before
	Biotinylated Sudan Black B (SBB) analog (GL13)	Antibody-enhanced detection
	Ubiquitin proteasome system	Eliminates damaged proteins
	Promyelocytic leukemia bodies	Reactive oxygen species and oxidative damage sensors
Extracellular vesicles	Extracellular vesicles	Increased secretion than before
	MicroRNA content of extracellular vesicles	Alterations in miRNA expression
Others	β-galactosidase	Increased production than before
	p53/p21	Increased expression than before
	P16INK4a	Increased expression than before
	Heterochromatin	Increased levels than before
	Urokinase plasminogen activator surface receptor	Up-regulation

### Senescence-associated β-galactosidase

Senescence-associated β-galactosidase (SA-β-gal, SABG) is one of the earliest described markers of senescence, which is used to detect senescent cell characteristics in senescence-related cells and tests.^38^ SA-β-gal staining is a simple colorimetric experiment that has been extensively applied in cell culture and tissue samples.^39^ Increased β-galactosidase activity indicates a positive histochemical staining result. The positive staining can be detected at a pH of 6.0 in senescent cells.^40^ Furthermore, SA-β-gal is typically not detected in prosenescent, quiescent, or transformed cells. Therefore, it can act as a biomarker for senescent cells. *In vivo*, positive senescent cells display differences in size and morphology compared with healthy cells, another feature of senescent cells detected by SA-β-gal.^41^ Cell enlargement may be related to intracellular metabolism alterations during the cellular stasis stage, resulting in pathological changes. Nonetheless, the precise mechanism remains unclear, necessitating further research.

However, the use of SA-β-gal as a marker for senescence has several limitations. SA-β-gal can accumulate in serum-starved cells and can serve as a part of the reversible response to mark specific macrophage subsets.^42^^,^^43^ SA-β-gal-positive cells have been observed in developing limbs during early embryonic stages.^44^ Studies have confirmed that these cells are non-senescent and undergoing apoptosis, with cell density potentially influencing SA-β-gal staining irrespective of the cell proliferation status.^45^ Moreover, basal autophagic activity increases following the isolation and culture of primary cells in monolayers, leading to a pronounced lysosomal response that produces SA-β-gal-positive staining at a pH of 6.0.^46^ Given that SA-β-gal is naturally present in lysosomes, it is unclear whether the SA-β-gal-positive staining observed in cultured chondrocytes signifies increased autophagy or actual cellular senescence. Despite its limitations, SA-β-gal remains the most widely used and important biomarker in cellular senescence-related research. A novel three-dimensional fluorescent probe, SA-HCy-1, capable of simultaneously detecting SA-β-gal, reactive oxygen species (ROS), and lysosomal pH, was developed to more accurately monitor the cellular senescence process.^47^

### P16

Another characteristic of senescent cells is the increased expression of cell cycle inhibitory proteins. Among these, p16^INK4a^ is considered a significant marker of cellular senescence. Senescent cells overexpress p16^INK4a^ while underexpressing genes encoding cell cycle-stimulating proteins. Conversely, p16^INK4a^ is infrequently expressed in young cells, making it a reliable marker for identifying senescent cells.^48^^,^^49^ Previous studies have demonstrated significant age-dependent differences in cyclin-dependent kinase inhibitor 2A (CDKN2A) expression in the ascending and descending colon of older adults compared with younger adults.^50^ Notably, the p16 protein is encoded by the CDKN2A gene. p16^INK4a^ is typically localized in the nucleus and functions as a cell cycle regulator by interacting with CDK4 to inhibit the G1/S transition.^51^ Furthermore, as a cyclin-dependent kinase inhibitor, p16^INK4a^ is an integral component of the p53 and RB-controlled tumor suppressor pathways and frequently accumulates in senescent cells.^52^ Numerous studies have employed p16^INK4a^ to identify senescent cells in tissues and cultured cells.

However, there are significant limitations to using p16^INK4a^ as a marker of cellular senescence *in vivo*. In certain contexts, senescent cells may not express p16^INK4a^ but instead up-regulate p15^INK4B^ or CDKN2C.^53^ Additionally, several studies have reported the presence of p16^INK4a^ in non-senescent cells, such as cancer cells, particularly those with inactivated Rb.^54^^,^^55^

### P53

Phosphorylated P53 is commonly utilized as a biomarker for cellular senescence. Following DNA double-strand breaks, cells initiate a stress response known as the DNA damage response. Unrepaired DNA damage is a key factor contributing to cellular senescence.^56^ Upon DNA double-strand break occurrence, ATM kinase is recruited to the site of damage, facilitating the assembly of specific DNA repair complexes through the phosphorylation of histone H2AX and histone methylation. Components include Kap-1, HP-1, and the H3K9 methyltransferase.^57^ This methylation event is followed by the activation of H3K9me3, which in turn activates the histone acetyltransferase Tip60, leading to the acetylation of ATM.^58^ The DNA repair mechanism necessitates the reversal of H3K9 methylation, but DNA damage response can also promote the degradation of G9a/GLP methyltransferases, resulting in reduced H3K9 dimethylation.^59^ Additionally, DNA damage response induces the phosphorylation of multiple serine residues on P53, enhancing P53’s transcriptional activity related to gene expression.^60^ Despite its utility, the use of phosphorylated P53 as a marker of cellular senescence has limitations. P53 expression is predominantly observed during the early stages of senescence and provides a protective effect that diminishes in the later stages of senescence. This temporal expression pattern suggests that phosphorylated P53 may not be a comprehensive marker for all stages of cellular senescence, necessitating cautious interpretation when used for senescence detection.

### P21

P21^WAF1/CIP1^ is a 21 kDa protein encoded by the CDKN1A gene, which belongs to the CDK1 Cip/Kip family, along with p27 and p57.^61^ This protein exhibits a dual role in cell cycle regulation. On one hand, p21 is essential for cell cycle progression. On the other hand, it can inhibit the kinase activity of cyclin-CDK complexes by interacting with cell cycle proteins through cyclin-binding motifs (Cy1 and Cy2).^62^ This inhibition prevents the phosphorylation of the RB protein family, resulting in the binding of RB to E2F and the formation of the DREAM complex, which ultimately inhibits cell cycle progression.^63^ Additionally, high levels of P21^WAF1/CIP1^ impede the formation of the D-CDK4/6 complex, further inhibiting cell cycle progression.^64^ P21 is up-regulated in response to senescence-inducing stimuli and acts downstream of p53, being largely regulated by the transcriptional activation of p53. Notably, p21 can also be activated by the tumor necrosis factor-beta (TNF-β) pathway in a p53-independent manner, with Sp1 acting as the primary transcription factor in this context.^65^^,^^66^ Overall, using p21 as a specific marker for cellular senescence is challenging due to its regulation by p53 and its involvement in other cellular processes.

### Emerging senescence markers

Several recent studies have been actively investigating novel markers of cellular senescence. A separate study identified and predicted hundreds of differentially expressed long non-coding RNAs (lncRNAs) and their targets in IDD. The results demonstrated that the up-regulation of lnc-HRK-2:1 might promote NP cell senescence, suggesting that lncRNAs could serve as robust indicators of cellular aging in NP cells.^67^ Meanwhile, proteomic analysis revealed that markers like phospholipase C gamma 1 (PLCG1) and stimulator of interferon (STING) may function as determinants of cellular senescence status.^68^ However, it should be noted that elevated PLCG1 levels are associated with dysfunctional chaperone-mediated autophagy. Additionally, a study reports that α-l-fucosidase exhibits significant up-regulation across various cellular senescence models.^69^ Therefore, a novel α-l-fucosidase-responsive fluorescent probe, QM-NHαfuc, was developed to enable real-time monitoring of cellular senescence both *in vitro* and *in vivo*, showing potential as a specific marker. Moreover, Amor C et al^70^ validated through flow cytometry that the urokinase plasminogen activator surface receptor (uPAR), a protein widely expressed on the surface of senescent cells, could serve as a distinctive marker of cellular senescence. As the understanding of aging markers and their roles in IDD continues to advance, more precise diagnostic tools can be anticipated.

### Cellular senescence in IDD

Recent investigations into cellular senescence in IDD have primarily focused on the senescence of NP cells. However, additional research on the senescence of AF cells and CEPs is warranted. Furthermore, understanding the mechanisms of cellular senescence in IDD is crucial for developing potential therapeutic approaches for managing disc degeneration.

### Senescence of nucleus pulposus

**Figure 2:**
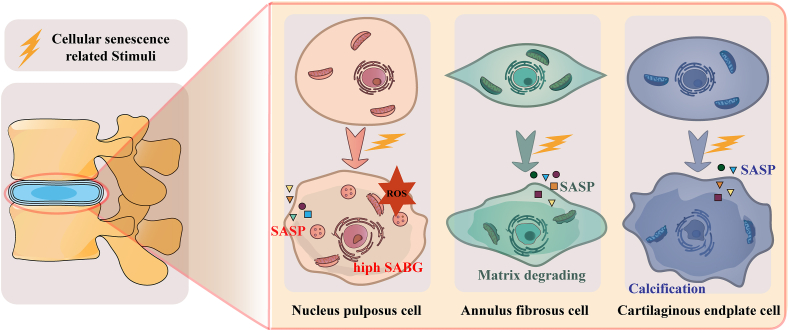
Morphological and pathological changes in intervertebral disc degeneration.

As IDD progresses, vacuolated and reticular-shaped NP cells disappear and are replaced by smaller, clustered cells within the cavity.^71^ These pathological small cells, formed by the fusion of original NP cell membranes, are commonly described as chondrocyte-like cells. The appearance of chondrocyte-like NP cells is indicative of disc degeneration.^72^ Increased fibrosis of NP cells, accompanied by reduced compliance, affects the biomechanics of the disc.^73^ The matrisome comprises various extracellular matrix proteins synthesized by cells in the intervertebral disc compartments to meet biomechanical requirements. The extracellular matrix, defined as the “matrisome”, accommodates cells and mediates cell–matrix interactions through changes in stiffness, thereby regulating downstream signaling activity and substantially influencing cell fate.^74^ Mounting evidence suggests that oxidative stress may be the initial causative factor of IDD, particularly as NP cells age.^75^^,^^76^ The accumulation of ROS leads to mitochondrial dysfunction and metabolic disorders, resulting in irreversible cell cycle arrest and cellular senescence (Fig. 2).^77^^,^^78^

Earlier studies have identified apoptosis as a major pathological change in IDD. GATA and forkhead box O (FOXO) are members of the transcription factor family that bind to specific DNA sequences to regulate the transcriptional activity of target genes.^79^^,^^80^ Previous studies have shown their involvement in regulating the cell cycle, DNA repair, and apoptosis. Recently, Wang et al^37^ reported that GATA4 expression was significantly up-regulated in acid-treated senescent NP cells, leading to cytokine release and down-regulation of the extracellular matrix through activation of the NF-κB pathway. Furthermore, GATA4 knockdown effectively alleviated stress-induced senescence in NP cells. However, the role of GATA4 in autophagy within NP cells remains unknown. Meanwhile, FOXO transcription factors, including FOXO1, FOXO3a, FOXO4, and FOXO6, are highly conserved protein kinase B (AKT) substrates.^81^ Multiple studies have demonstrated that aberrant deactivation of FOXO contributes to age-related diseases such as cancer and diabetes.^82^ Alvarez-Garcia et al^83^ have observed that FOXO plays a critical role in the progression of IDD. Importantly, FOXO-regulated genes are involved in the cell cycle, apoptosis, DNA damage response, and aging.^84^ As a central regulator of IVD homeostasis, FOXO directly governs autophagy in NP cells by conferring cellular resistance against oxidative and inflammatory stimuli.^85^ Following oxidative stress, phosphorylation of FOXO1 is increased, accompanied by a reduction in sirtuin 1 (SIRT1) nuclear translocation, thereby mitigating cellular senescence.^86^

**Figure 3:**
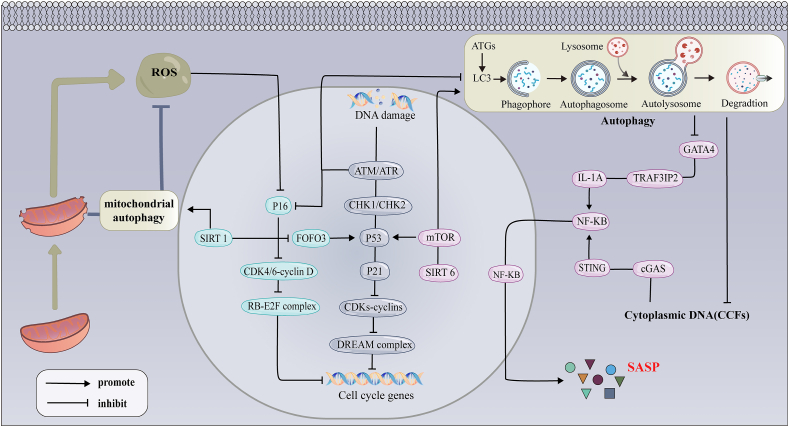
Mechanisms of cellular senescence and the senescence-associated secretory phenotype (SASP) in intervertebral disc degeneration.

In recent years, numerous studies have investigated the relationship between autophagy and cellular senescence mechanisms in IDD, with particular emphasis on mitochondrial autophagy (mitophagy).^87^^,^^88^ Nowadays, autophagy has been demonstrated to prevent premature senescence under distinct conditions in IDD^89^, ^90^, ^91^; however, the relationship between senescence and autophagy is intricate. Autophagy is a cellular degradation process that maintains intercellular homeostasis under physiological conditions and serves as a cytoprotective mechanism.^92^ Stress conditions activate autophagy-related genes (ATGs), driving structural changes in subcellular membranes that encapsulate cytoplasmic components, eventually resulting in the formation of autophagosomes.^93^ Following their fusion with lysosomes, the encapsulated cargoes within autophagosomes undergo gradual degradation, a process known as autophagic flux.^94^ The microtubule-associated protein 1 light chain 3 (LC3) is a ubiquitinated protein existing in two forms: the cytosolic form LC3-I and the phosphatidylethanolamine-bound form LC3-II. LC3-II is a protein marker reliably associated with autophagosome maturation.^95^ The expression levels of LC3-II and beclin-1 are decreased in human degenerate NP cells, suggesting disruptions in the initiation of autophagy. Autophagy is regulated by the mammalian target of rapamycin (mTOR), a serine/threonine kinase involved in cell growth and division.^96^ The mTOR complex 1 (mTORC1) inhibits the phosphorylation of AKT by class-I phosphatidylinositol 3-kinase (PI3K), which in turn inhibits apoptosis and promotes cell survival through a negative feedback loop involving p70 ribosomal S6 kinase (p70/S6K) (Fig. 3).^97^

DNA damage is a major cause of senescence stress. Following DNA damage, histone γ-H2AX undergoes phosphorylation.^98^ Damaged DNA fragments can penetrate the cytoplasm and are recognized by the cyclic GMP-AMP synthase (cGAS)-STING pathway, which activates the NF-κB signaling pathway.^99^ Subsequently, NF-κB signaling phosphorylates and facilitates the translocation of P65 to the nucleus, promoting inflammatory cytokine production and triggering the generation of SASP, ultimately leading to cellular senescence.^100^^,^^101^ Activation of autophagy degrades damaged DNA fragments and down-regulates γ-H2AX levels, consequently inhibiting the activation of the cGAS-SYING pathway and reducing pro-inflammatory responses.^102^ This reduction in inflammatory factor release inhibits SASP, resulting in the suppression of cellular senescence.

Sirtuins are a family of NAD^+^-dependent histone deacetylases consisting of SIRT1–7. Notably, they protect against age-related diseases such as neurodegeneration, osteoporosis, and cardiovascular disease.^103^^,^^104^ SIRT1, in particular, is crucial for cell survival and is implicated in various cellular processes, including cell cycle regulation, metabolism, and senescence.^105^, ^106^, ^107^ It has been widely acknowledged for its protective role against NP degeneration through multiple pathways during IDD progression. In patients with disc degeneration, the expression of SIRT1 and lactate dehydrogenase A (LDHA) is down-regulated, leading to increased cellular senescence.^108^ Furthermore, the up-regulated expression of SIRT1 alleviates cellular senescence by activating mitochondrial autophagy, resulting in the removal of damaged mitochondria and the inhibition of ROS accumulation.^109^, ^110^, ^111^ In addition, SIRT1 activation maintains NP cell morphology by inhibiting the NF-κB inflammatory pathway.^112^

SIRT2 acted as a protective protein to diminish senescent NP cells by suppressing the P53/P21 pathway and oxidative stress in severely degenerated discs.^113^ Meanwhile, SIRT3 regulates mitochondrial function and contributes to the pathogenesis of IDD. Previous studies have highlighted that the expression levels of SIRT3 in degenerative NP tissues are significantly lower than those in healthy humans and rats.^114^ In contrast, overexpression or activation of SIRT3 protected rat NP cells from oxidative stress-induced senescence by limiting ROS accumulation through superoxide dismutase 2 (SOD2) deacetylation.^115^ Additionally, in H_2_O_2_-induced and tert-butyl hydroperoxide (TBHT)-induced premature senescence of NP cells, SIRT3 was involved in regulating the AMP-activated protein kinase (AMPK)/peroxisome proliferator-activated receptor-gamma coactivator 1 (PGC-1) signaling pathway.^116^

SIRT6, another member of the sirtuin family, mediates senescence in various pathological processes, with its expression down-regulated in senescent NP cells.^117^ Past studies have determined that activation of SIRT6 inhibits senescence by enhancing autophagy via mTOR and inhibiting NF-κB to promote extracellular matrix metabolism in NP cells.^118^^,^^119^ However, the regulation of NP cell senescence by other members of the sirtuin family has not been identified so far.

Additionally, heme oxygenase-1 (HO-1) has been described as protective against disease progression in various pathological conditions due to its antioxidant, anti-inflammatory, and anti-aging effects.^120^^,^^121^ Overexpression of HO-1 significantly reduces mitochondrial membrane potential, ROS accumulation, and mitochondrial morphological changes, thereby attenuating senescence and apoptosis in NP cells during IDD.^122^^,^^123^ This effect is achieved by promoting mitochondrial autophagy and concomitantly inhibiting mitochondrial dysfunction. Furthermore, the mitogen-activated protein kinase (MAPK) is also closely related to autophagy and cellular senescence in NP cells. It has been suggested that the activation of the MAPK/NF-κB signaling pathway worsens IDD-associated phenotypes by activating the extracellular matrix degeneration pathway in NP cells.^124^^,^^125^

### Senescence of annulus fibrosus

Various studies have demonstrated significant age-related changes in the AF, including alterations in cell morphology, extracellular matrix composition, and mechanical properties, which collectively contribute to degenerative disc disease.^126^^,^^127^ Therefore, it is essential to explore the senescence of AF cells and elucidate their role in disc degeneration. Recent investigations have elucidated that ROS precipitates senescence in AF cells, contributing to disc degeneration and associated pathologies.^128^ Nonetheless, the specific impact of AF cell senescence on disc degenerative diseases has yet to be fully delineated. Zhong et al^129^ have demonstrated that AF cells exhibit senescence in response to ionizing radiation, which in turn up-regulates matrix-degrading enzymes, notably matrix metalloproteinase (MMP)-1 and MMP-3 proteins. Moreover, the process of AF cell senescence appears to be modulated by autophagy mechanisms.

A key investigation revealed the critical role of SIRT2, a marker of the sirtuin family, in safeguarding cells against ROS damage. Within the nucleus, SIRT2 deacetylates histone H4 at lysine 16 (H4K16), a modification that influences cell cycle regulation.^130^ Additionally, SIRT2 deacetylates FOXO3a, thereby activating the transcription of SOD2 gene, which encodes the mitochondrial superoxide dismutase (MnSOD) protein, a potent antioxidant.^131^^,^^132^ Notably, SIRT2 is implicated in the regulation of mitochondrial biogenesis through modulation of PGC-1α and up-regulation of antioxidant enzymes, thereby diminishing ROS levels.^133^ Xu et al^134^ report that SIRT2 modulates the level of oxidative stress-induced mitochondrial autophagy via PGC-1α, thereby mitigating IDD.

Furthermore, recent research has identified that inhibition of mTORC1-induced autophagy via the PI3K/Akt/mTOR pathway, contingent upon Akt and mTORC2 activity, alleviates the adverse effects of AF cells in disc cells.^97^ While several studies have focused on the role of AF cells in disc degeneration, there has been relatively limited exploration into the significance of autophagy in the senescence of these cells.

### Senescence of cartilaginous endplate

The CEPs, which separate the disc from adjacent vertebrae, play a critical role in nutrient exchange and mechanical support. As the largest avascular tissue in the body, CEPs facilitate the diffusion of nutrients from the peripheral vascular system to the IVDs and the removal of waste from the IVDs.^135^ Additionally, CEPs provide spinal flexibility while playing a crucial mechanical role in preventing the disc from bulging out of the foramina.^136^ The thickness of CEPs varies significantly with age, spinal location, IVD location, and tissue region. During disc degeneration, endplate chondrocytes are subjected to oxidative stress and inflammatory insults, leading to the generation of ROS and the release of pro-inflammatory cytokines.^137^ These mediators perturb chondrocyte homeostasis, augment extracellular matrix degradation, and contribute to the calcification of the CEP, thus exacerbating the progression of IDD.^138^ Antioxidative agents have been shown to mitigate CEP calcification.

Ferroptosis functions as a form of non-apoptotic cell death that depends on iron.^139^ Elevated levels of ferroptosis were observed in chondrocyte degeneration and IDD compared with healthy counterparts.^140^ Glutathione peroxidase 4 (GPX4) is a key regulator of ferroptosis, and its activity is impaired during aging.^141^ Under inflammatory conditions, Piezo1 is up-regulated and participates in the regulation of CEP cell senescence and apoptosis by activating the Ca^2+^/CaMKII/Drp1 axis.^142^ In senescent cells, inhibition of Piezo1 channel activity increased GPX4 expression and attenuated ferroptosis phenotype.^143^ Furthermore, transferrin receptor-1 (TFR1) serves as the primary cellular iron gate that mediates iron homeostasis and is also crucial for ferroptosis and aging.^144^ The nuclear factor erythroid 2-related factor 2 (Nrf2) signaling pathway, a crucial component of the cellular defense mechanism against oxidative stress, orchestrates the induction of mitophagy to curtail ferroptosis.^145^ This regulatory mechanism attenuates CEP degeneration and the advancement of IDD by inhibiting apoptotic pathways, osteogenic differentiation, and the up-regulation of extracellular matrix-degrading enzymes.^146^ Meanwhile, autophagy can also reverse CEP apoptosis and calcification via the SIRT1 autophagy.^147^

Recent studies have demonstrated that the ROS/p38-MAPK/NF-κB signaling pathway can regulate EPC senescence and calcification through oxidized low-density lipoproteins (ox-LDL)/lectin-like oxidized low-density lipoprotein receptor-1 (LOX-1), suggesting that dyslipidemia may contribute to IDD by influencing cellular senescence.^148^ However, the effects of cellular senescence on endplate cells remain underexplored. Further research is imperative to explicate the impact of autophagy and cellular senescence on the pathogenesis of CEP degeneration in the context of IDD.

### Senescence inhibition: A potential therapy for IDD

Recent studies have indicated that senolytics and senomorphics could alleviate aging-related disorders in mammals.^149^ Given the critical role of cellular senescence in the progression of IDD and disc maintenance, targeting senescence-associated pathways represents a promising therapeutic approach for IDD. Senolytics have been shown to directly induce apoptosis in senescent cells, thereby locally eliminating abnormal pathological conditions.^150^ Conversely, senomorphics aim to suppress SASPs and prevent SASP components from affecting healthy cells in the microenvironment and converting them into senescent cells.^151^ This strategy helps to prevent the further deterioration of aging-related diseases (Table 2).

**Table 2:** Senolytics and senomorphics: Potential drug candidates for intervertebral disc degeneration.

Drug name	Targets	Administration	Effects on intervertebral disc degeneration	Reference
*Senolytics*				
Dasatinib + quercetin	Multiple tyrosine kinases	Intraperitoneal injections (5 mg/kg dasatinib plus 50 mg/kg quercetin weekly)	Targets survival-promoting networks, such as tyrosine kinases, BCL-2, p53, p21, serine, and PI3K/AKT, to influence the cell cycle	176
Quercetin	Multiple signaling	Intragastric administration (100 mg/kg every other day)	Inhibits IL-1β-induced cascade activation of the NF-κB pathway	177
			Binds to the Keap1-Nrf2 complex, thereby inhibiting the NF-κB pathway	
		Cell treatment (60 μM)	Promotes SIRT1-dependent autophagy	178
		Cell treatment (20 μM)	Reduces oxidative stress through the miR-34a/SIRT1 signalling pathway	159
UBX0101	MDM2/p32	___	___	163
RG-7112	MDM2/p32	Cell treatment (5 μM)	Down-regulates INF-γ, IL-6, CCL24, CXCL1, CXCL10, and angiogenin	161
o-Vanillin	MDM2/p53	Cell treatment (100 μM)	Down-regulates CCL2,5,7,8, GM-CSF, BDNF, NGF, TNF-α, CLCX1, CLCX8, CLCX10, IL-1β, and IL-8 expression	179
RG-7112 + o-Vanillin	MDM2/p53	Cell treatment (5 μM of RG-7112 and 100 μM of o-Vanillin)	Down-regulates IL-6, IL-8, IL-1β, and TNF-α expression	162
Fisetin	PI3K/AKT/mTOR	Cell treatment (20 μM)	Mediates through the Nrf2/HO-1 axis	180
		Cell treatment (10 μM)	May mediate oxidative stress via SIRT1	181
A-1331852 A-1155463	BCL-XL (prosurvival protein)	___	___	182,183
ABT-737/ABT-263 (navitoclax)	BCL-2, BCL-XL, and BCL-W (prosurvival proteins)	___	___	184
Cardiac glycosides (including oubain and digoxin)	BCL-2, BCL-XL, and BCL-W (prosurvival proteins)	Intraperitoneal injections (50 nM concentration of digoxin twice weekly)	Inhibits TNF-α-induced inflammation	185
			Attenuates extracellular matrix destruction	
			Significantly promotes extracellular matrix anabolism	
*Senomorphics*				
Metformin	IKK and/or NF-κB	Intraperitoneal injections (50 mg/kg body weight/day)	Possibly inhibits senescence through autophagy inactivation of the cGAS-STING pathway	102
	AMPK	Cell treatment (100 μM)	Activates autophagy in an AMPK-dependent pathway	171
Periostin neutralizing antibodies	NF-κB	___	Blocks the PIEZO1-induced self-amplification cycle of NF-κB and periostin	186
BAY 11-7082	NF-κB p65 subunit and IκB	Cell treatment (10 μM)	Suppresses NF-κB	187
Rapamycin	mTOR	Cell treatment (15 μM)	Inhibits mTORC1 via the PI3K/Akt/mTOR pathway to induce autophagy	97
		Cell treatment (25 μM)	Decreases the gene expression of MMP-3, MMP-13, IL-1β, IL-6, TNF-α, and protein levels of P16 and P21	126
RAD001 (everolimus)	mTOR	____	Decreases p70/S6K but increases Akt phosphorylation and promotes autophagy	174,188
Lutikizumab	IL-1α, IL-1β	____	____	189
KU-60019	ATM	____	____	190,191
Ruxolitinib	JAK1/2	____	____	192

### Senolytics for IDD

Previous studies have demonstrated that senolytic drugs can eliminate senescent cells and effectively extend the lifespan of mice. Two viable senolytic agents were initially considered: dasatinib and quercetin. Dasatinib, a multi-tyrosine kinase inhibitor, interferes with ephrin B-dependent apoptosis, reducing the viability and inducing cell death in senescent cells.^152^ Quercetin, a natural flavonol, inhibits the phosphatase and tensin homolog (PTEN)/PI3K/AKT signaling pathway, reducing the viability of senescent cells.^153^

The combination of dasatinib and quercetin has shown a broader targeting of senescent cell types *in vitro*. Zhu et al^154^ identified an amplified pro-survival network expression in senescent cells, which aligns with their ability to evade apoptosis. This drug combination selectively induces apoptosis in senescent preadipocytes and endothelial cells. Furthermore, the combination of dasatinib and quercetin has been used as a senolytic in various age-related diseases, including idiopathic pulmonary fibrosis and bone loss, yielding beneficial outcomes.^155^^,^^156^ In the context of chondrocytes, the combination demonstrates significant therapeutic efficacy, reducing pain and inflammation in aged animals and increasing cartilage development in young animals.^157^ Additionally, the combined drugs selectively target senescent cells in mice, thereby alleviating age-related diseases.^158^

Recent research breakthroughs have further elucidated the mechanisms of these two drugs. A study established that quercetin can reduce oxidative stress-induced senescence of nucleus pulposus mesenchymal stem cells via the miR-34a/SIRT1 signaling pathway, exerting time-dependent effects towards the restoration of cell health.^159^ This finding underscores the potential of quercetin in mitigating cellular senescence through targeted molecular pathways.

RG-7112, a member of the nutlin family, was developed as an MDM2 inhibitor for cancer treatment by stabilizing p53 and enhancing its anti-cancer activity.^160^ Recent studies indicate that RG-7112 can selectively eliminate senescent intervertebral disc cells, particularly senescent AF and NP cells.^161^ *In vitro*, RG-7112 exerts therapeutic effects on human IVD cells by inducing apoptosis through the activation of the caspase-3 pathway.^161^^,^^162^ This effect is comparable to that of UBX0101, which exacerbates senescent chondrocyte apoptosis in a mouse model of osteoarthritis.^163^ Phase I and II clinical trials are currently underway to evaluate the safety, tolerability, and clinical efficacy of UBX0101 in osteoarthritis patients with moderate-to-severe pain, involving single-dose (NCT03513016 and NCT04129944) and repeat-dose (NCT04229225) administration. However, the effectiveness of UBX0101 in delaying IVD degeneration remains unestablished.

Previous studies have shown that senescent cells up-regulate anti-apoptotic mediators, such as members of the B-cell lymphoma 2 (BCL-2) family (including BCL-2, BCL-W, and BCL-XL).^164^ Based on this mechanism, senolytic agents, such as ABT-737 and ABT-263 (navitoclax), have been used to inhibit BCL-2 family activity, leading to the apoptosis of senescent cells.^165^^,^^166^ Additionally, compounds like ouabain, EF24, and proxofim have demonstrated selective reduction of senescent cell activity. Ouabain, a cardiac glycoside analog, promotes apoptosis by inducing the BCL-2 family protein NOXA.^167^ EF24 facilitates proteasomal degradation of BCL-2, while proxofim disrupts the binding of p53 to FOXO4.^168^ A-1331852 and A-1155463, which inhibit BCL-XL, have also been investigated in senescent chondrocytes.^169^ Proxofim, a peptide, promotes apoptosis by interfering with the p53-FOXO4 interaction, effectively killing senescent cells or reducing their activity.^170^

These drugs hold potential for the treatment of IVD degeneration. However, further *in vivo* and *in vitro* studies are necessary to evaluate their efficacy and safety in this context.

### Senomorphics for IDD

The therapeutic targeting of pathways and molecules associated with inflammation and disease is a well-established strategy. Senomorphics have been employed to inhibit molecules associated with SASP. Numerous senomorphic candidates, including inhibitors of inhibitory kappa B (IκB) kinase (IKK), NF-κB, mTOR (such as rapamycin), ATM (such as KU-60019), and inhibitor of TNF-α (INF; such as etanercept), have been shown to exert anti-senescence effects without inducing apoptosis. Furthermore, the activation of platelet-derived growth factor (PDGF) and fibroblast growth factor (FGF) signaling pathways has demonstrated promising outcomes in mitigating senescence-related processes.

Classical inflammatory modulators associated with NF-κB activity can modulate SASP. Several studies have demonstrated the efficacy of agents that modulate the NF-κB signaling pathway, such as metformin and BAY 11–7082, in decreasing SASP production. Metformin is commonly used as a hypoglycemic drug and has been shown to extend the lifespan of individuals with type II diabetes. Additionally, it is one of the most extensively studied senomorphic drugs for delaying the onset of age-related diseases. Previous studies have reported that metformin induces autophagy to degrade damaged DNA fragments in the cytoplasm of senescent NP cells.^171^ This effect impedes the activation of the cGAS-STING and NF-κB signaling pathways, resulting in a decrease in the levels of pro-inflammatory factors, which in turn suppresses the secretion of SASP.^102^ In senescent AF cells, metformin inhibited catabolic production and cell senescence by blocking the HMGB1 translocation.^172^ The PI3K/Akt/mTOR signaling pathway is pivotal in regulating the fate, survival, and stromal homeostasis of cells under inflammatory stress.^173^ Rapamycin, a well-known senomorphic agent, inhibits mTORC1, thereby mitigating inflammation and cellular senescence by suppressing the release of pro-inflammatory SASP factors through the induction of autophagy.^126^ In rabbit AF cell cultures, rapamycin inhibits the phosphorylation of mTOR and its downstream effector p70/S6K. However, for rapamycin to exert its therapeutic effects on AF cells by attenuating inflammatory responses via mTOR-mediated autophagy, the activation of Akt must remain unaffected.^97^ Some studies have demonstrated that RAD001, an analog of rapamycin, exhibits similar inhibition of mTOR phosphorylation. Nonetheless, cell cycle arrest and SA-β-Gal positivity persisted, potentially due to RAD001 down-regulating the phosphorylation of IKKβ and promoting the degradation of IL-1α receptor-associated kinase 1 (IRAK1) and IκBα following DNA damage. Notably, these responses were reversed by exogenous IL-1α.^174^^,^^175^

Collectively, these findings suggest that the inhibition of SASP via senomorphics may be a promising therapeutic strategy for the treatment of IDD. However, further research is required to elucidate the precise contribution of SASP to IDD progression and the potential of senomorphic interventions.

### Perspective

The precise mechanisms through which cellular senescence contributes to IDD remain incompletely understood. Emerging evidence suggests that persistent cellular senescence occurs in intervertebral disc tissues under various risk factors. Cellular senescence may play a pivotal role in the initiation and progression of IDD. Furthermore, the accumulation of senescent cells in IDD can cause significant damage, leading to severe degenerative changes.

In studies investigating the senescence phenotype in disc cells, it is imperative to identify and establish markers that can accurately quantify senescence status. However, the currently utilized senescence markers present several limitations that need to be addressed. One of their principal limitations is their lack of specificity for cellular senescence, which can result in inaccurate assessments of cellular senescence status. SA-β-gal activity, as well as the expression of p16 and p21, is commonly employed as markers of senescence. However, it is important to note that these markers may be present in non-senescent disc cells and can be affected by factors other than senescence. SA-β-gal staining may not be a definitive indicator of disc cell senescence, given that it is influenced by changes in autophagy and lysosomal function.

To address these limitations, current research efforts should aim to identify and validate novel, specific, sensitive, and reliable markers that comprehensively and accurately reflect cellular senescence status.

Senescent cells have been detected in various intervertebral disc cell populations, including NP cells, AF cells, and endplate cells, using available markers. These senescent cells exhibit altered gene expression patterns, increased secretion of pro-inflammatory cytokines, and enhanced production of matrix-degrading enzymes. These changes collectively disrupt IVD homeostasis and initiate degenerative processes. Previous studies have emphasized that NP cell senescence contributes to pathological changes during IDD. Additionally, several signaling pathways have been identified as key mediators of senescence in NP cells, and the role of oxidative stress-mediated autophagy in NP cell senescence has also been investigated.

To comprehensively elucidate the effects of aging on disc degeneration, additional research should focus on cellular senescence in both AF and EP cells, which are critical for maintaining the structural integrity and function of intervertebral discs. AF cells provide mechanical support and preserve the structural integrity of the IVD. Consequently, senescent AF cells can undermine the structural integrity of the AF, leading to IDD. EP cells, on the other hand, supply nutrients and oxygen to the avascular IVDs. Thus, senescent EP cells may impair nutrient transport and reduce metabolic activity, contributing to disc degeneration. Understanding the role of EP cell senescence may offer new insights into the mechanisms underlying disc degeneration.

The interaction of each component in IVD tissues is crucial for maintaining homeostasis. Considering their intricate relationships, previous studies have pioneered new therapeutic strategies and interventions to alleviate the burden of degenerative disc disease on patients. Further investigation is needed to identify the precise mechanisms by which senescence induces IDD phenotypes. The IVD is a complex structure containing various cell types, and evidence suggesting a specific cell type as the primary driver of IDD is lacking. Assessing the specificity of drugs for IDD treatment is also challenging. Anti-senescence drugs typically target a specific mechanism, eliminate senescent cells, or regulate metabolism, but a single target may not be sufficient to fully reverse or halt the degeneration. In addition, the non-specific effects caused by candidate drugs with potential for clinical treatment, such as possible liver and kidney toxicity or immune disorders, also urgently need attention. Meanwhile, IVD is an avascular structure in the human body, and developing drugs that accurately target the inside of the disc and maintain sufficient concentration is a technical bottleneck. We considered whether blood vessel neovascularization with leaky vessels in the degenerative process could be expected to help in drug delivery. However, anti-aging drugs have shown promise in the treatment of other diseases. Overall, there is a pressing need to investigate the relationship between various cell types and cellular senescence in IDD to provide reliable and credible conclusions that can inform subsequent clinical interventions for IDD.

## Conclusion

In conclusion, this review comprehensively outlined the molecular and cellular mechanisms implicated in cellular senescence and critically appraises the merits and limitations of methodologies extensively utilized in prior investigations to determine the initiation of cellular senescence. Furthermore, this review synthesizes contemporary research elucidating the contributions of cellular senescence within the NP, AF, and CEPs of intervertebral disc components, indicating prospective avenues for forthcoming research endeavors. Lastly, this review catalogs senotherapeutic agents with anti-aging effects for IDD and evaluates their prospective impact on future clinical therapeutic research.

## CRediT authorship contribution statement

**Yunbo Guan:** Writing – review & editing, Software, Writing – original draft, Visualization. **Xuedong Bai:** Writing – original draft, Writing – review & editing. **Chao Li:** Writing – original draft, Writing – review & editing, Investigation. **Ziqiang Zhang:** Writing – review & editing. **Qing He:** Writing – review & editing. **Lin Chen:** Formal analysis, Software, Writing – review & editing, Visualization. **Yangli Xie:** Investigation, Project administration, Writing – review & editing, Supervision. **Zuqiang Wang:** Conceptualization, Funding acquisition, Writing – review & editing, Writing – original draft.

## Funding

This work was supported by the 10.13039/100014717National Natural Science Foundation of China (No. 82102594).

## Conflict of interests

The authors declared no competing interests.
